# GPER Mediates a Feedforward FGF2/FGFR1 Paracrine Activation Coupling CAFs to Cancer Cells toward Breast Tumor Progression

**DOI:** 10.3390/cells8030223

**Published:** 2019-03-07

**Authors:** Maria Francesca Santolla, Adele Vivacqua, Rosamaria Lappano, Damiano Cosimo Rigiracciolo, Francesca Cirillo, Giulia Raffaella Galli, Marianna Talia, Giuseppe Brunetti, Anna Maria Miglietta, Antonino Belfiore, Marcello Maggiolini

**Affiliations:** 1 Department of Pharmacy, Health and Nutritional Sciences, University of Calabria, 87036 Rende, Italy; mariafrancesca.santolla@unical.it (M.F.S.); adele.vivacqua@unical.it (A.V.); rosamaria.lappano@unical.it (R.L.); damianorigiracciolo@yahoo.it (D.C.R.); francesca.cirillo@unical.it (F.C.); giulia.r.galli@gmail.com (G.R.G.); mariannatalia11@gmail.com (M.T.); 2 University of Natural Resources and Life Sciences, 1180 Vienna, Austria; giusep.bru@gmail.com; 3 Regional Hospital, 87100 Cosenza, Italy; annamariamiglietta@virgilio.it; 4 Endocrinology, Department of Clinical and Experimental Medicine, University of Catania, Garibaldi-Nesima Hospital, 95122 Catania, Italy; antonino.belfiore@unict.it

**Keywords:** cancer-associated fibroblasts, GPER, breast cancer, estrogen, FGFR1, FGF2

## Abstract

The FGF2/FGFR1 paracrine loop is involved in the cross-talk between breast cancer cells and components of the tumor stroma as cancer-associated fibroblasts (CAFs). By quantitative PCR (qPCR), western blot, immunofluorescence analysis, ELISA and ChIP assays, we demonstrated that 17β-estradiol (E2) and the G protein estrogen receptor (GPER) agonist G-1 induce the up-regulation and secretion of FGF2 via GPER together with the EGFR/ERK/c-fos/AP-1 signaling cascade in (ER)-negative primary CAFs. Evaluating the genetic alterations from METABRIC and TCGA datasets, we then assessed that FGFR1 is the most frequently amplified FGFRs family member and its amplification/expression associates with shorter survival rates in breast cancer patients. Therefore, in order to assess the functional FGF2/FGFR1 interplay between CAFs and breast cancer cells, we generated the FGFR1-knockout MDA-MB-231 cells using CRISPR/Cas9 genome editing strategy. Using conditioned medium from estrogen-stimulated CAFs, we established that the activation of FGF2/FGFR1 paracrine signaling triggers the expression of the connective tissue growth factor (CTGF), leading to the migration and invasion of MDA-MB-231 cells. Our findings shed new light on the role elicited by estrogens through GPER in the activation of the FGF2/FGFR1 signaling. Moreover, our findings may identify further biological targets that could be considered in innovative combination strategies halting breast cancer progression.

## 1. Introduction

Cross-talk between stromal and epithelial cells plays an important role in diverse pathophysiological conditions, including malignant diseases [1,2,3]. In this regard, it has been largely reported that the acquisition of an aggressive phenotype does not depend exclusively on the intrinsic cancer cell properties, but also on stromal features [4]. For instance, cancer-associated fibroblasts (CAFs), one of the most abundant cell types within the tumor microenvironment, coordinate a multifaceted biochemical program that promotes cancer cell proliferation, migration, invasion, epithelial-mesenchymal transition (EMT), and angiogenesis [5]. Indeed, it has been shown that paracrine mediators secreted by CAFs, such as cytokines and growth factors, exert an important role in the acquisition of malignant features [6,7].

The fibroblast growth factor (FGF)-FGF receptor (FGFR) axis is one of the major signal transduction pathways mediating the interaction between tumor stroma and cancer cells [8,9]. FGFRs family includes four highly conserved transmembrane receptor tyrosine kinases (FGFR1-4) and one receptor that binds to FGF ligands, although it lacks the intracellular kinase domain (FGFR5, also known as FGFRL1) [10]. FGFRs can be activated either in an autocrine fashion by FGFs produced by the tumor cells or in a paracrine manner by FGFs secreted by the stromal components [11]. The activation of FGFRs triggers the phosphorylation of extracellular signal-regulated kinase (ERK), phosphatidylinositol 3-kinase (PI3K), and other transduction pathways, regulating many physiological processes including embryogenesis, tissue homeostasis, and angiogenesis [12,13]. Abnormal activation of the FGFR1-mediated signaling pathway can be caused by translocation, point mutation and amplification of the FGFR1 gene, hence leading to malignant transformation and cancer progression [9,14,15]. Likewise, increased FGF2 levels have been observed in plasma samples of patients affected by diverse malignancies, such as leukemia and lung and breast cancers, especially when metastases are present [16,17]. Among diverse stimuli, FGF2 expression and secretion can be regulated by estrogens [18,19], which act mainly through the classical estrogen receptors (ER)α and ERβ leading to the proliferation, migration and survival of breast cancer cells [20]. The G protein estrogen receptor (GPER, also called GPR30) has been identified as a further receptor able to mediate the action of estrogens in numerous pathophysiological conditions [21,22]. GPER activation induces a network of signal transduction pathways including activation of the epidermal growth factor receptor (EGFR), intracellular cyclic adenosine monophosphate (AMP) accumulation, calcium mobilization, activation of ERK1/2 and PI3K [23]. Moreover, GPER triggers the expression of various genes involved in the growth and migration of diverse estrogen-responsive tumors [24,25,26,27]. Of note, the stimulatory action mediated by estrogenic GPER signaling has been also evidenced in breast primary CAFs revealing the existence of a functional cooperation between these important components of the tumor stroma and cancer cells [28,29,30,31]. Recent studies have shown that a functional interaction between ER and FGFR-mediated pathways may occur toward breast cancer progression, indicating that the simultaneous inhibition of both receptors could be considered in more comprehensive treatments [11,32,33]. GPER was also involved in the estrogen-induced regulation of FGF2 toward the autocrine stimulation of the cognate receptor FGFR1 in astroglial cells [19].

Here, we provided novel insights into the ability of estrogens to regulate a feedforward FGF2/FGFR1 activation between the ER-negative CAFs and breast cancer cells. On the basis of our findings, GPER may be included among the factors facilitating the estrogen-activated cross-talk within the tumor microenvironment toward breast tumor progression.

## 2. Materials and Methods

### 2.1. Reagents

We purchased (1-[4-(-6-bromobenzol [1,3] diodo-5-yl)-3a,4,5,9b-tetrahidro3H5cyclopenta[c]quinolin-8yl]-ethanone) (G-1), (3aS,4R,9bR)-4-(6-Bromo-1, 3-benzodioxol-5-yl)-3a,4,5,9b-3H-cyclopenta[c]quinolone (G15) from Tocris Bioscience (Space, Milan, Italy); 17β-Estradiol (E2), Wortmannin (WM) from Sigma-Aldrich (Milan, Italy); PD173074 from Selleckchem (DBA, Milan, Italy); PD98059 from Calbiochem (DBA, Milan, Italy); tyrphostin AG1478 from Biomol Research Laboratories (Milan, Italy) and recombinant human Fibroblast Growth Factor (FGF2) 100-18B, from PEPROTECH (SIAL, Rome, Italy). All compounds were solubilized in dimethyl sulfoxide (DMSO), except for FGF2, which was dissolved in aqueous buffer (0.1% BSA).

### 2.2. Cell Cultures

MDA-MB-231, MCF-7, and SkBr3 breast cancer cells were obtained from the ATCC (Manassas, USA). MDA-MB-231 and MCF-7 cells were maintained in DMEM/F12 (Life Technologies, Milan, Italy), 10% fetal bovine serum (FBS) and 1% of penicillin/streptomycin, while SkBr3 cells were maintained in RPMI-1640 (Life Technologies, Milan, Italy), 10% fetal bovine serum (FBS), and 1% of penicillin/streptomycin (Life Technologies, Milan, Italy). Cells were used less than six months after resuscitation and mycoplasma negativity was tested monthly. CAFs were extracted from invasive mammary ductal carcinomas obtained from mastectomies, while normal fibroblasts (NFs) were isolated from a non-cancerous breast tissue at least 2 cm from the outer tumor margin, as previously described [25,28,34]. Primary cells cultures of breast fibroblasts were characterized by immunofluorescence. Cells were incubated with anti-vimentin (V9, sc-6260), anti-cytokeratin 14 (LL001 sc-53,253) and anti-fibroblast activated protein α (FAPα) (H-56) antibodies that were obtained from Santa Cruz Biotechnology (DBA, Milan, Italy) (Supplementary Figure S1). All cells were grown in a 37 °C incubator with 5% CO_2_ and switched to medium without serum and phenol red the day before treatments to be processed for immunoblot and quantitative PCR (qPCR) assays.

### 2.3. Gene Expression Studies

Total RNA was extracted and complementary DNA (cDNA) was synthesized by reverse transcription as described in our previous work [35]. Quantitative PCR (qPCR) assays were performed using platform Quant Studio7 Flex Real-Time PCR System (Life Technologies, Milan, Italy). Gene-specific primers were designed using Primer Express version 2.0 software (Applied Biosystems). For FGF2, FGFR1, c-fos, CTGF and the ribosomal protein 18S, which was used as a control gene to obtain normalized values, the primers were: 5′-AGTGTGTGCTAACCGTTACCT-3′ (FGF2 forward) and 5′-ACTGCCCAGTTCGTTTCAGTG-3′ (FGF2 reverse); 5′- CCCGTAGCTCCATATTGGACA-3′ (FGFR1 forward) and 5′- TTTGCCATTTTTCAACCAGCG-3′ (FGFR1 reverse); 5′-CGAGCCCTTTGATGACTTCCT-3′ (c-fos forward) and 5′-GGAGCGGGCTGTCTCAGA-3′ (c-fos reverse); 5′-ACCTGTGGGATGGGCATCT-3′ (CTGF forward) and 5′-CAGGCGGCTCTGCTTCTCTA-3′ (CTGF reverse); 5′- GGCGTCCCCCAACTTCTTA-3′ (18S forward) and 5′-GGGCATCACAGACCTGTTATT-3′ (18S reverse). Assays were performed in triplicate and the results were normalized with control mRNA levels of 18S. Relative mRNA levels were calculated using the ^ΔΔ^Ct method comparing to control group.

### 2.4. Gene silencing Experiments and Plasmids

Cells were transfected by X-treme GENE 9 DNA Transfection Reagent (Roche Diagnostics, Sigma-Aldrich, Milan, Italy) for 24 h before treatments with a control vector and a specific shRNA sequence for each target gene. The short hairpin (sh)RNA constructs to knock down the expression of GPER and CTGF, and the unrelated shRNA control constructs have been described previously [24,30,36]. The plasmid DN/c-fos, which encodes for c-fos mutant that heterodimerizes with c-fos dimerization partners but does not allow DNA binding, was a kind gift from Dr C. Vinson (NIH, Bethesda, MD, USA).

### 2.5. CRISPR/Cas9-Mediated FGFR1 Knockout

Short guide RNA (sgRNA) sequence targeting human FGFR1 was designed using the E-CRISP sgRNA Designer (http://www.e-crisp.org/E-CRISP/) and cloned into the pSpCas9 (BB)-2A-Puro (PX459) vector (kindly provided by Dr. W.T. Khaled, University of Cambridge, UK) according to the protocol described in Ran et al. [37]. The *FGFR1* sgRNA sequence is as follows: *sgFGFR1*: 5′-CGGCCTAGCGGTGCAGAGTG-3′. Then, the plasmid with sgRNA was transiently transfected into MDA-MB-231 cells using Lipofectamine LTX (Life Technologies, Milan Italy). Two days after transfection the cells were selected via growth in a medium contained 1 µg/mL puromycin dihydrochloride (Sigma-Aldrich, Milan, Italy). After puromycin selection, the puromycin-resistant colonies were picked and expanded in regular medium. Then, immunoblots for FGFR1 protein were performed to evaluate the efficiency of the FGFR1 knockout.

### 2.6. Immunofluorescence Microscopy

50% percent confluent cultured grown on coverslips were serum deprived for 24 h and then treated for 6 h with E2 and G-1 alone and in combination with G15, as indicated. Where required, cells were previously transfected for 24 h with shRNA or shGPER (as described above) and then treated for 6 h with E2 and G-1. Next, cells were fixed in 4% paraformaldehyde in PBS, permeabilized with 0.2% Triton X-100, washed 3 times with PBS and incubated at 4 °C overnight with a mouse primary antibody against FGF2 (C-2) (Santa Cruz Biotechnology, DBA, Milan, Italy). After incubation, the slides were extensively washed with PBS, probed with donkey anti-mouse IgG-FITC (1:300; purchased from Alexa Fluor, Life Technologies, Milan Italy) and 4, 6-diamidino-2-phenylindole dihydrochloride (DAPI) (1:1000; Sigma-Aldrich, Milan, Italy). Then, the images were obtained using the Cytation 3 Cell Imaging Multimode reader (BioTek, AHSI, Milan Italy) and analyzed by the Gen5 software (BioTek, AHSI, Milan Italy).

### 2.7. Conditioned Medium Derived from CAFs

CAFs were cultured in regular growth medium, then washed twice with PBS and transferred in medium without serum for 24 h. Next, CAFs were treated for 6 h with E2 and G-1 alone and in combination with G15, as indicated; then, cells were washed twice with PBS and cultured for additional 12 h with fresh serum-free medium. Thereafter, the supernatants were collected, centrifuged at 3500 rpm for 5 min to remove cell debris and used as conditioned medium in the appropriate experiments.

### 2.8. Western Blot Analysis

CAFs and MDA-MB-231 cells were processed to obtain protein lysates for western blot analysis as previously described [29]. Primary antibodies were as follows: GPER (AB137479) (Abcam, Euroclone Milan, Italy); CTGF (TA806803) (OriGene Technologies, DBA, Milan, Italy); FGFR1 (#9740) and p-FGFR1 (#3476) (CST, Euroclone Milan, Italy); c-fos (E8), phosphorylated extracellular signal-regulated kinase (ERK) (E-4), ERK2 (C-14), p-AKT1/2/3 (Ser 473)-R, AKT/1/2/3 (H-136) and β-actin (AC-15) (Santa Cruz Biotechnology, DBA, Milan, Italy). Proteins were detected by horseradish peroxidase-linked secondary antibodies (Biorad, Milan, Italy) and revealed using the chemiluminescent substrate for western blotting Westar Nova 2.0 (Cyanagen, Biogenerica, Catania, Italy).

### 2.9. Enzyme-Linked Immunosorbent Assay

The concentrations of FGF2 in conditioned medium collected from CAFs were measured using human FGF2 ELISA Kit (Thermo Fisher Scientific, Monza Italy), according to the manufacturer’s instructions. Briefly, after incubation with conditioned media for 2 h at room temperature (RT), the plates were washed four times using 1X wash buffer, then 100 μL Hu FGF2 Biotin conjugate solution were added into each well, except the chromogen blanks, for 1 h at RT. Next, the plates were washed four times using 1X wash buffer and then 100 μL 1X Streptavidin-HPR solution were added into each well for 30 min at RT. Following incubation, plates were washed four times using 1X wash buffer and a colour-substrate solution was added to each well. After incubation in the dark for 30 min at RT, 100 μL of stop solution was used to stop reaction. Then, the plates were read at 450 nm on a Microplate Spectrophotometer Epoch™ (BioTek, AHSI, Milan Italy).

### 2.10. Chromatin Immunoprecipitation (ChIP) Assay

Chip experiments were performed as previously described [29]. The primers used to amplify a region containing an AP-1 site located into the FGF2 promoter sequence were: 5′-GTTTCTACAAGGAGGCACGTC-3′ (Fw) and 5′-GAGATGCCAAATCTGATGCCA-3′ (Rv). qPCR data were normalized respect to unprocessed lysates (Input DNA) and the results were reported as fold changes respect to nonspecific IgG.

### 2.11. Analysis of Public Datasets from METABRIC and TCGA and Kaplan-Meier Plotter

Images of genomic alterations in Molecular Taxonomy of Breast Cancer International Consortium (METABRIC) and The Cancer Genome Atlas (TCGA) databases were captured from cBioPortal (http://www.cbioportal.org) [38,39]. Prognostic values of mRNA expression or copy-number (CN) gains of FGFR1 from METABRIC [40,41] breast cancer samples were analyzed by Kaplan-Meier survival curves. Long-rank test was used for statistical analysis. The mRNA expression z-Scores of FGFR1 and CTGF were retrieved from METABRIC [40,41] breast cancer samples analyzed for gene expression using Illumina Human v3 microarray. Data were processed using the Python programming language to identify correlation patterns among different genes. In particular, pairwise linear regressions of mRNA levels between FGFR1 and CTGF were calculated. The Pearson correlation coefficients measured the magnitude of the linear relationship between genes.

### 2.12. Polarization Assay

FGFR1 (WT) and FGFR1 (KO) MDA-MB-231 cells were serum deprived for 24 h and then exposed for 8 h to conditioned media collected from CAFs or to FGF2, as indicated. Then cells were processed as previously described [28,42].

### 2.13. Scratch Assay

FGFR1 (WT) and FGFR1 (KO) MDA-MB-231 cells were seeded into 12-well plates and they were allowed to grow in regular growth medium until they were 70–80% confluent. Next, cells were switched in medium without serum and after 24 h a p200 pipette tip was used to create a scratch of the cell monolayer. Cells were washed twice with PBS and then incubated at 37 °C with conditioned media collected from CAFs or with FGF2 for 24 h, as indicated. Pictures were photographed at 0 h and 24 h after scratching using inverted phase contrast microscope (5×magnification). The rate of cell migration was calculated by quantifying the % of wound closure area using the WCIF ImageJ software, according to the formula:

% of wound closure = [(At = 0 h) – (At = Δ h)/(At = 0 h)] × 100%

### 2.14. Transwell Migration and Invasion Assays

Transwell 8 µm polycarbonate membrane (Costar, Sigma-Aldrich, Milan, Italy) was used to evaluate in vitro migration and invasion of FGFR1 (WT) and FGFR1 (KO) MDA-MB-231 cells. 5 × 10^4^ cells in 300 μL serum-free medium were seeded in the upper chamber, coated with (invasion assay) or without (migration assay) Corning^®^ Matrigel^®^ Growth Factor Reduced (GFR) Basement Membrane Matrix (Biogenerica, Catania, Italy) at a 1:3 dilution. Medium containing 10% FBS was then added into the lower chamber as a chemoattractant. 4 h after seeding, cells on the upper surface of the membrane were removed by wiping with Q-tip, and invaded or migrated cells were fixed with 100% methanol, stained with Giemsa (Sigma-Aldrich, Milan, Italy), photographed using Cytation 3 Cell Imaging Multimode Reader (BioTek, AHSI, Milan Italy) and counted using the WCIF ImageJ software.

### 2.15. Statistical Analysis

Data were analyzed by one-way ANOVA with Dunnett’s multiple comparisons, where applicable, using GraphPad Prism, 6.01 (GraphPad Software, Inc., San Diego, CA, USA). (*) *p* < 0.05 and (**) *p* < 0.01 were considered statistically significant.

### 2.16. Ethics Approval and Consent to Participate

All procedures are conformed to the Helsinki Declaration for the research on humans. Signed informed consent was obtained from all patients and the experimental research has been performed with the ethical approval provided by the “Comitato Etico Regione Calabria, Cosenza, Italy” (approval code: 166, 2 December 2016).

## 3. Results

### 3.1. GPER Mediates the Induction of FGF2 Expression by E2 and G-1 in Breast Cancer-Associated Fibroblasts (CAFs)

**Figure 1:**
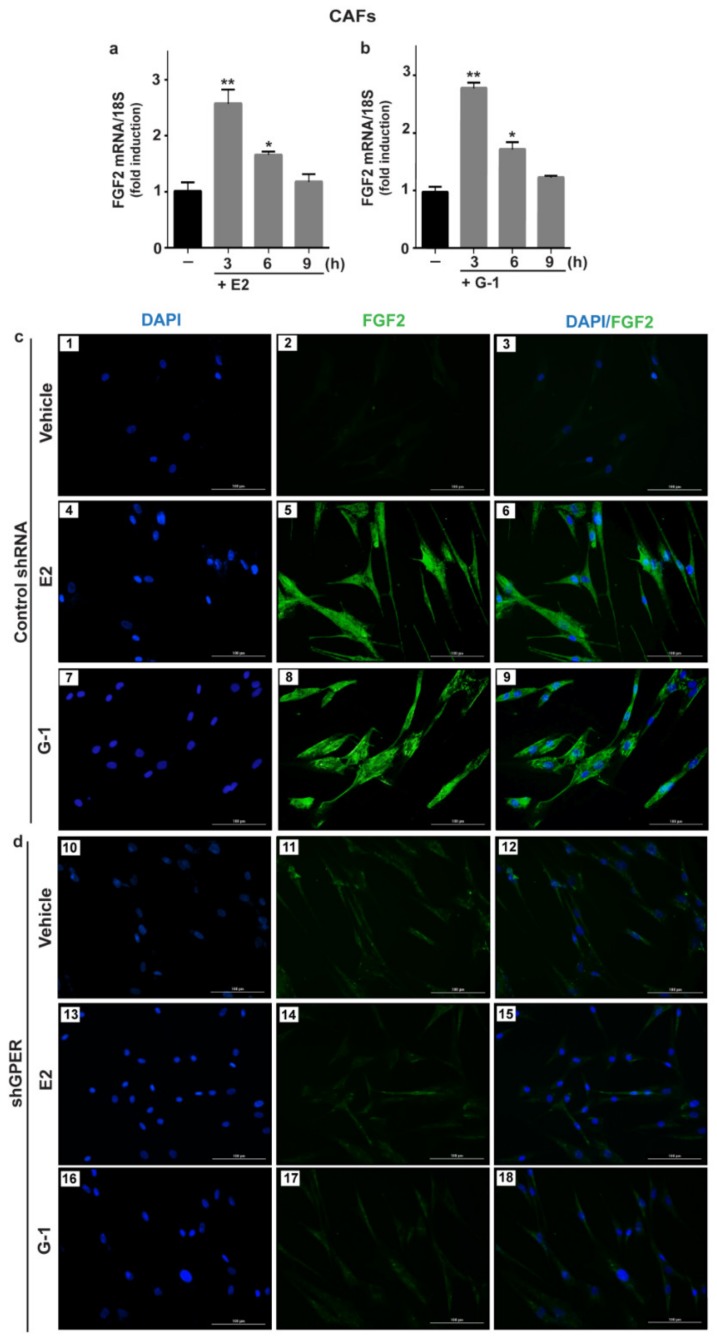
E2 and G-1 induce FGF2 expression through GPER in CAFs. 10 nM E2 (**a**) and 100 nM G-1 (**b**) induced FGF2 mRNA expression, as evaluated by quantitative PCR (qPCR). Values were normalized to 18S expression and shown as fold changes of FGF2 mRNA expression upon E2 and G-1 treatments respect to cells exposed to vehicle (). Each column represents the mean ± standard deviation (SD) of three independent experiments performed in triplicate. (**) indicates *p* < 0.01 and (*) indicates *p* < 0.05. (**c**,**d**) FGF2 protein expression by immunofluorescence in CAFs transfected for 24 h with control shRNA (panels 1–9) or sh G protein estrogen receptor (shGPER) (panels 10–18) and then treated for 6 h with vehicle, 10 nM E2 and 100 nM G-1, as indicated. FGF2 accumulation is shown by the green signal, nuclei are stained by 4, 6-diamidino-2-phenylindole dihydrochloride (DAPI) (blue signal), scale bar = 100 μm. Images shown are representative of two independent experiments.

**Figure 2:**
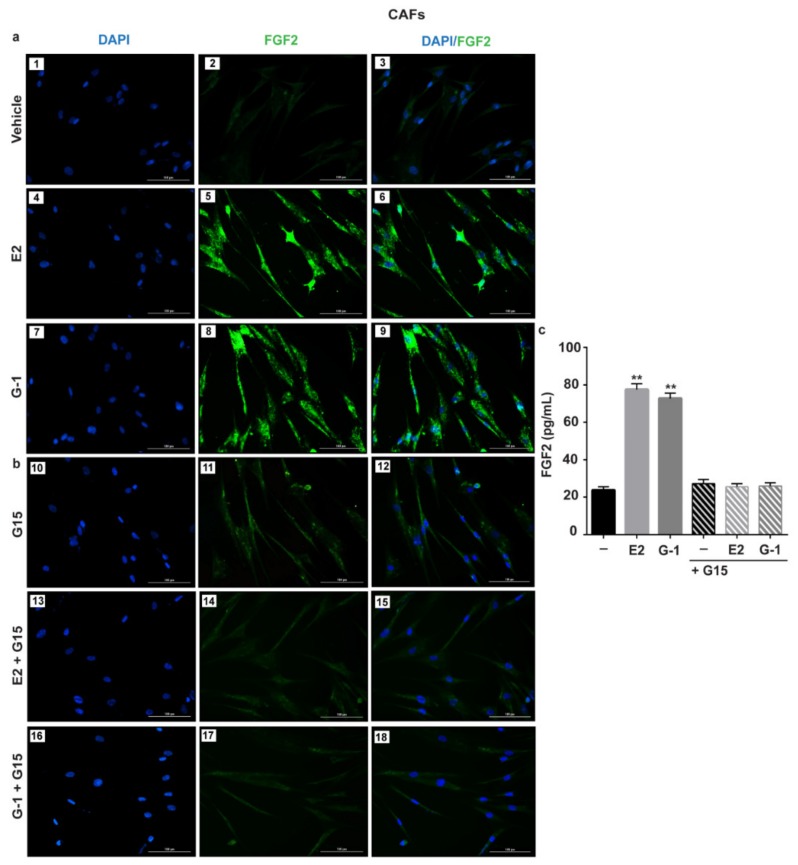
GPER mediates the up-regulation and the secretion of FGF2 by E2 and G-1 in CAFs. FGF2 protein expression by immunofluorescence in CAFs treated for 6 h with vehicle, 10 nM E2 and 100 nM G-1, alone (panels 1–9) (**a**) and in combination with 100 nM GPER antagonist G15 (panels 10–18) (**b**). FGF2 accumulation is shown by the green signal, nuclei are stained by DAPI (blue signal), scale bar = 100 μm. Images shown are representative of two independent experiments. (**c**) ELISA of FGF2 levels in supernatants collected from CAFs treated for 18 h with vehicle (-), 10 nM E2 and 100 nM G-1 alone and in combination with 100 nM GPER antagonist G15. Each column represents the mean ±SD of three independent experiments performed in triplicate. (**) indicates *p* < 0.01.

Previous studies have shown that estrogens acting either through ER or GPER up-regulate FGF2 expression and secretion in both normal and cancer cells [19,32,43]. In order to provide novel insights into the FGF2 regulation by estrogens within the tumor microenvironment, we sought to address whether estrogens may regulate FGF2 levels in ER-negative/ GPER-positive CAFs isolated from breast tumor patients (see material and methods section). Worthy of note, both E2 and G-1 induced the expression of FGF2 at the mRNA (Figure 1a,b) and protein levels (Figure 1c) in CAFs. However, the response to E2 and G-1 was no longer observed after GPER silencing (Figure 1d, Supplementary Figure S2) or using the GPER antagonist G15 (Figure 2a,b). In contrast, E2 and G-1 were not able to elicit FGF2 up-regulation in fibroblasts derived from noncancerous breast tissue (data not shown). By performing ELISA experiments, we then observed that the secretion of FGF2 in CAFs medium upon treatments with E2 and G-1 is abrogated treating cells with the GPER antagonist G15 (Figure 2c). As GPER activation induces the stimulation of diverse transduction pathways [23], we also found that FGF2 up-regulation prompted by E2 and G-1 was prevented either by the EGFR tyrosine kinase inhibitor AG1478 (AG) or the MEK inhibitor PD98059 (PD), but not by the PI3K inhibitor Wortmannin (WM) (Supplementary Figure S3a,b). Taken together, these findings indicate that, in CAFs, both E2 and G-1 induce FGF2 expression through the GPER-EGFR-ERK1/2 signaling cascade.

### 3.2. c-fos is Involved in the FGF2 up-Regulation Induced by Estrogenic GPER Signaling in CAFs

**Figure 3:**
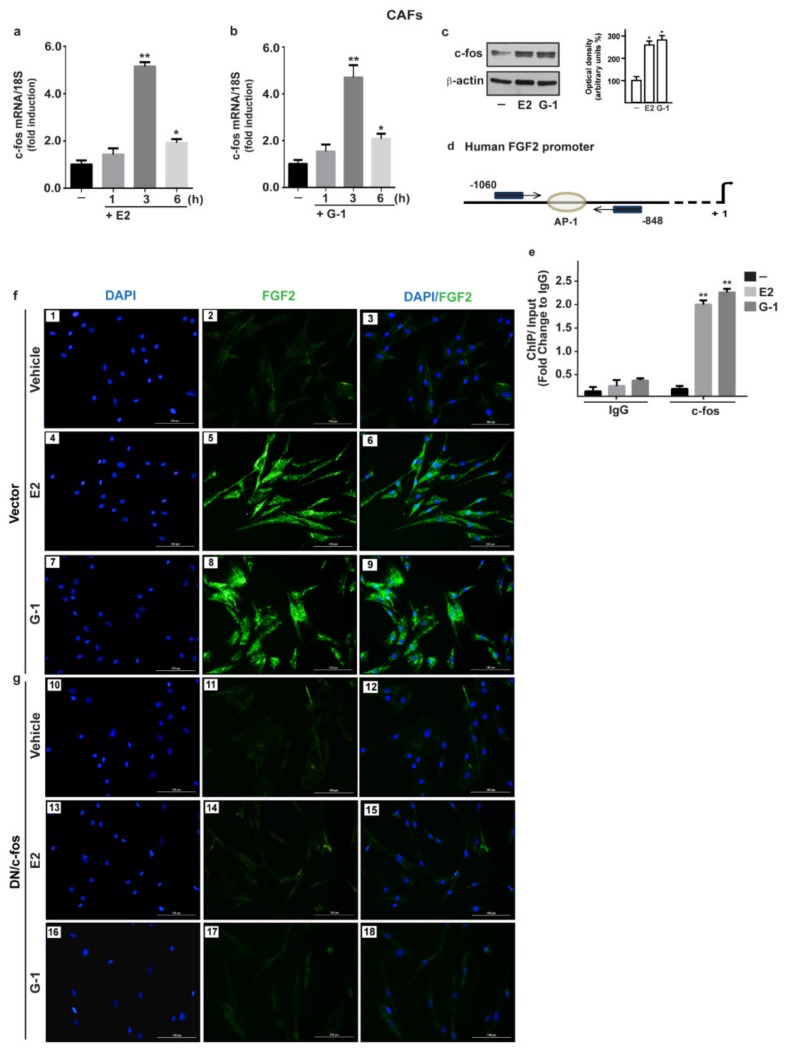
The oncogene fos (c-fos) is involved in the up-regulation of FGF2 by E2 and G-1 in CAFs. 10 nM E2 (**a**) and 100 nM G-1 (**b**) induced c-fos mRNA expression, as evaluated by qPCR. Values were normalized to 18S expression and shown as fold changes of c-fos mRNA expression upon E2 and G-1 treatments respect to cells exposed to vehicle (-). (**c**) The treatment for 3 h with 10 nM E2 and 100 nM G-1 up-regulated c-fos protein, which is recruited to the AP-1 site located within the FGF2 promoter region (-1060/-848; the transcriptional start site is indicated as + 1), as ascertained by Chromatin Immunoprecipitation (ChIP)-qPCR assay (**d**,**e**). Data were normalized to the input and reported as fold changes respect to Immunoblobulin G (IgG). Each column represents the mean ±SD of three independent experiments performed in triplicate. In immunoblot experiments β-actin served as a loading control, side panels show densitometric analysis of the blot normalized to the loading control. (*) indicates *p* < 0.05 and (**) indicates *p* < 0.01. (**f**) FGF2 protein expression by immunofluorescence in CAFs transfected for 18 h with a vector (panels 1–9), or (**g**) with a construct encoding for a dominant negative form of c-fos (DN/c-fos) (panels 10–18) and then treated for 6 h with vehicle, 10 nM E2 and 100 nM G-1, as indicated. FGF2 accumulation is evidenced by the green signal, nuclei are stained by DAPI (blue signal), scale bar = 100 μm. Images shown are representative of two independent experiments.

As the activation of GPER-EGFR-ERK1/2 transduction pathway leads to c-fos expression [22,29], we determined c-fos response at both mRNA and protein levels upon E2 and G-1 exposure in CAFs (Figure 3a–c). Then, we established that both ligands trigger the recruitment of c-fos to the AP-1 site located within the FGF2 promoter region (Figure 3d,e). Further supporting these results, the up-regulation of FGF2 protein expression induced by E2 and G-1 was prevented transfecting CAFs with a dominant negative form of c-fos (DN/c-fos) (Figure 3f,g). Collectively, the abovementioned findings suggest that, in CAFs, GPER along with the EGFR-ERK1/2-c-fos-AP-1 signaling pathway mediates FGF2 expression in response to E2 or G-1.

### 3.3. Conditioned Medium (CM) from Estrogens-Stimulated CAFs Activates the FGFR1-ERK1/2-AKT Transduction Pathway in MDA-MB-231 Cells

**Figure 4:**
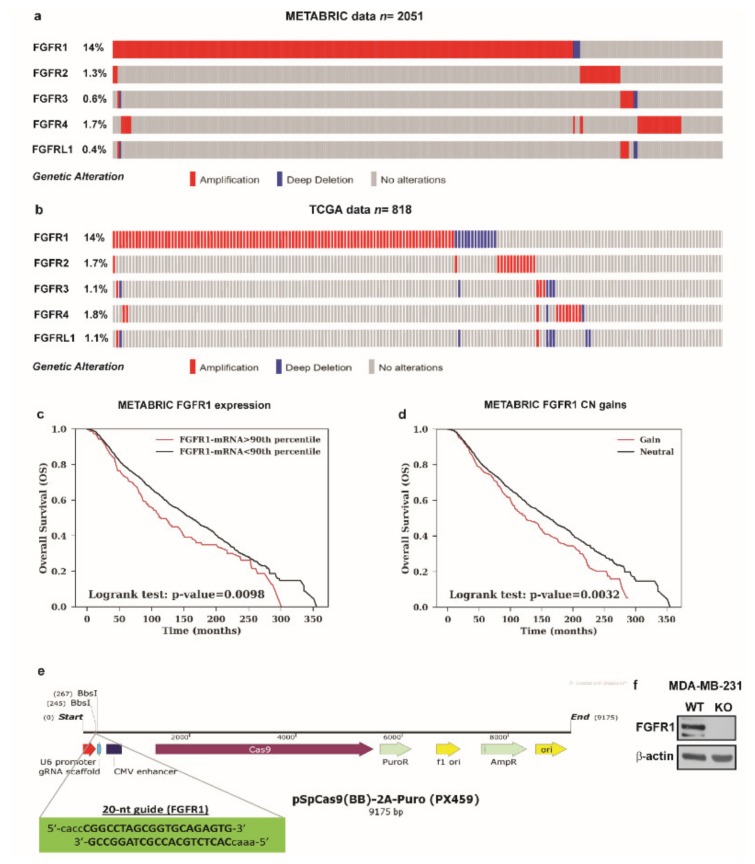
Analysis of METABRIC and TCGA datasets and CRISPR/Cas9-mediated FGFR1 knockout (KO) in MDA-MB-231 cells. (**a**,**b**) The OncoPrint of genomic alteration of FGFRs members showed that FGFR1 is the most amplified receptor of the family in human breast cancer patients. Each row represents a gene and each column represents a tumor sample. Red bars indicate gene amplifications, blue bars deep deletions and grey bars no alterations. (**c**,**d**) Kaplan-Meir plots show the overall survival (OS) from METABRIC dataset between patients with normal or high FGFR1 mRNA expression or between patients with copy number (CN) gains or without (neutral). Statistical analysis was performed using the long-rank test. (**e**) Schematic representation of the pX459 plasmid and the sgRNA sequence used to generate FGFR1 (KO) MDA-MB-231 cells. (**f**) Immunoblots of lysates generated from FGFR1 (WT) and FGFR1 (KO) MDA-MB-231 cells. β-actin served as loading control. Immunoblots shown are representative of three independent experiments.

**Figure 5:**
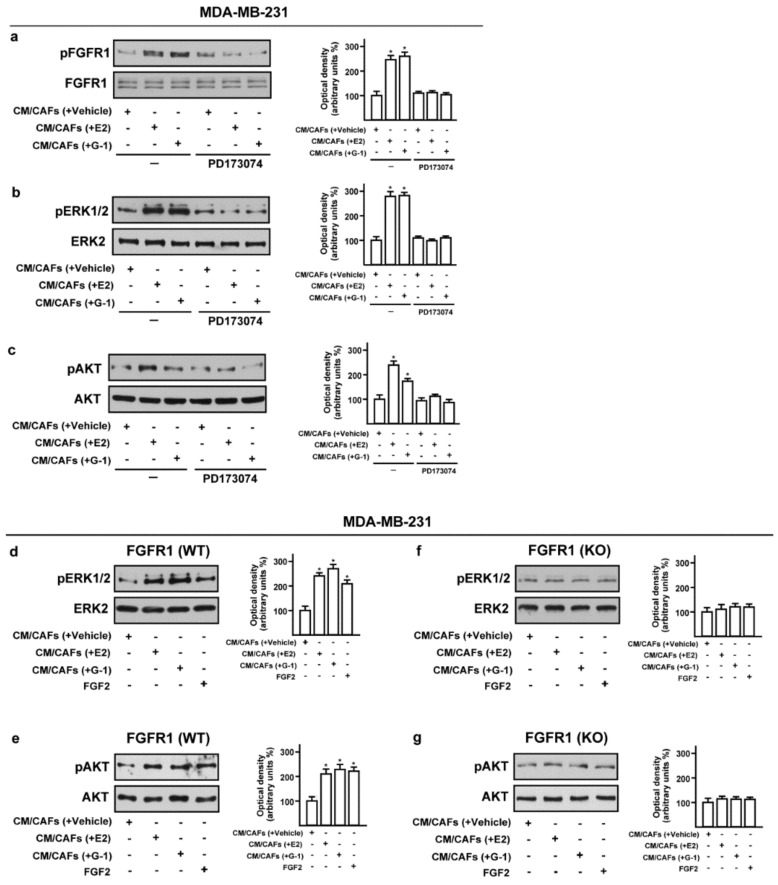
Conditioned medium (CM) from estrogen-stimulated CAFs induces the activation of FGFR1- signaling pathway in MDA-MB-231 cells. (**a**–**c**) Phosphorylation of FGFR1, ERK1/2, AKT in MDA-MB-231 cells exposed for 1 h to CM from CAFs treated for 18 h with vehicle [CM/CAFs (+vehicle)], 10 nM E2 [CM/CAFs (+E2)] or 100 nM G-1 [CM/CAFs (+G-1)], alone and in the presence of 1 μM FGFR1 inhibitor PD173074. (**d**,**e**) Activation of ERK1/2 and AKT in FGFR1 (WT) MDA-MB-231 cells upon exposure for 1 h to CM from CAFs treated for 18 h with 10 nM E2 [CM/CAFs (+E2)], 100 nM G-1 [CM/CAFs (+G-1)]; (**f**,**g**) In FGFR1 (KO) MDA-MB-231 cells cultured in the same conditions as described above, the activation of ERK1/2 and AKT was no longer observed. FGF2 at 25 nM was used as positive control. FGFR1, ERK2, AKT and β-actin served as loading control, as indicated. Side panels show densitometric analysis of the blots normalized to the loading controls. Immunoblots shown are representative of three independent experiments. (*) indicates *p* < 0.05.

Previous studies have shown that the activation of the FGF2-FGFR1 autocrine and/or paracrine loop plays an important role toward the migration and invasion of cancer cells [44,45,46]. Moreover, FGFR1 has been found highly amplified in breast cancer patients and associated with endocrine resistance [11,14]. In accordance with these observations, a large-scale genomic analysis of METABRIC and TCGA databases allowed us to assess not only that FGFR1 represents the most amplified receptor of the FGFRs family members but also that FGFR1 amplification occurs in nearly 14% of breast cancer patients (Figure 4a,b) [38,39]. Of note, breast cancer patients with either higher expression or copy number (CN) gains of FGFR1 are associated with shorter overall survival rates respect to the rest of the cohort (Figure 4c,d). Taken into account the aforementioned results, we focused on FGF2-FGFR1 signaling in the context of paracrine communication between CAFs and breast cancer cells. In this vein, we used MDA-MB-231 cells as model system because these cells did not express FGF2 [47], but rather displayed high expression levels of FGFR1 (Supplementary Figure S4). In order to better investigate the role of FGFR1 in our experimental model, we used CRISPR/Cas9 genome editing technology to generate FGFR1 knockout (KO) MDA-MB-231 cell line (Figure 4e,f). Given that both E2 and G-1 stimulate the expression and the secretion of FGF2 in CAFs (see Figure 1; Figure 2), we then ascertained that CM from E2 and G-1 treated-CAFs induces FGFR1 phosphorylation in MDA-MB-231 cells, as well as the stimulation of the two main pathways downstream FGFR1 activation, such as ERK1/2 and AKT [11,48] (Figure 5a–c). Next, in parallel experiments, the FGFR1 inhibitor PD173074 was found to abolish both ERK1/2 and AKT phosphorylation (Figure 5a–c). Accordingly, CM obtained from E2 and G-1 treated-CAFs did not induce ERK1/2 and AKT activation in FGFR1 (KO) MDA-MB-231 cells (Figure 5d–g).

### 3.4. FGF2/FGFR1 Paracrine Activation Up-Regulates CTGF Expression in MDA-MB-231 Cells

**Figure 6:**
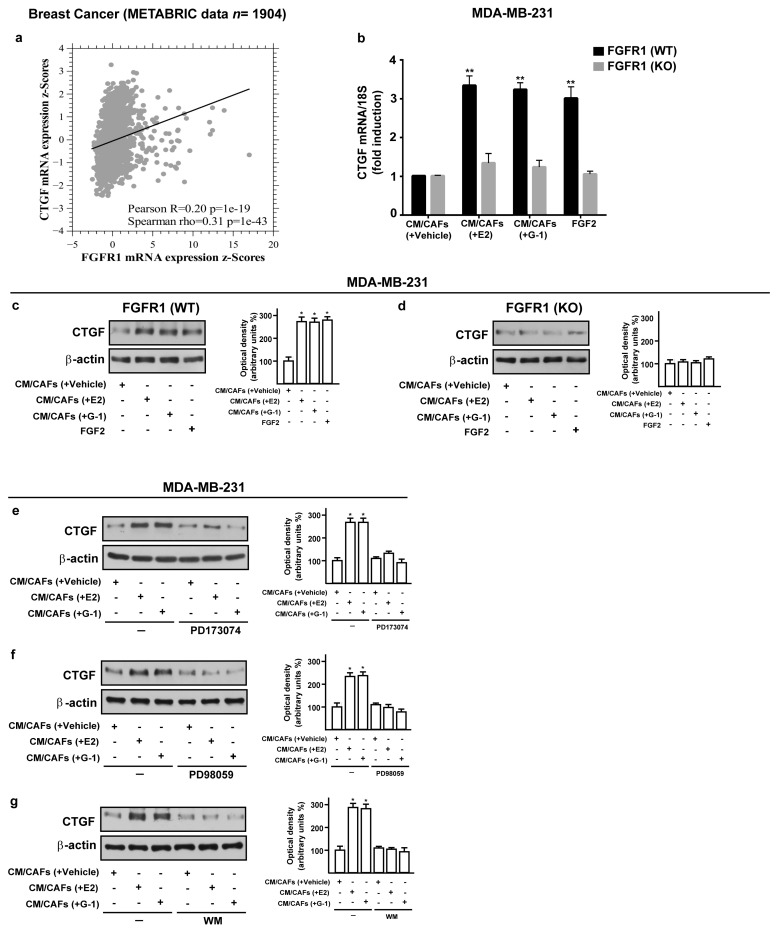
Conditioned medium (CM) from estrogen-stimulated CAFs up-regulates CTGF levels through FGFR1 signaling pathway in MDA-MB-231 cells. (**a**) Pairwise linear regressions of FGFR1 versus CTGF mRNA levels were performed on METABRIC dataset of 1904 breast tumor samples. Scatter plot shows positive correlation between FGFR1 and CTGF expression. (**b**–**d**) CTGF mRNA and protein levels in FGFR1 (WT) and FGFR1 (KO) MDA-MB-231 cells exposed for 3 h to CM from CAFs treated for 18 h with vehicle [CM/CAFs (+vehicle)], 10 nM E2 [CM/CAFs (+E2)] or 100 nM G-1 [CM/CAFs (+G-1)], or exposed to 25 nM FGF2, as positive control, evaluated by qPCR and western blot. In RNA experiments, values were normalized to the expression of 18S and shown as fold changes of CTGF mRNA expression upon CM from CAFs treated with E2 and G-1 respect to cells exposed to CM from CAFs treated with vehicle. Each column represents the mean ±SD of three independent experiments performed in triplicate. (**e**–**g**) Up-regulation of CTGF protein expression in MDA-MB-231 cells exposed for 3 h to CM from CAFs treated for 18 h with vehicle [CM/CAFs (+vehicle)], 10 nM E2 [CM/CAFs (+E2)], or 100 nM G-1 [CM/CAFs (+G-1)] was no longer observed in the presence of 1 μM FGFR1 inhibitor PD173074, 10 μM MEK inhibitor PD98059 or 100 nM PI3K inhibitor Wortmannin (WM). β-actin served as loading control. Side panels show densitometric analysis of the blots normalized to the loading controls. Immunoblots shown are representative of three independent experiments. (**) indicates *p* < 0.01 and (*) indicates *p* < 0.05.

A synergic action between FGF2 and connective tissue growth factor (CTGF) may occur in diverse pathophysiological conditions [49,50,51]. Hence, we analyzed the mRNA levels of FGFR1 and CTGF in METABRIC breast cancer patients database [40,41] and we found a positive correlation between FGFR1 and CTGF expression (Figure 6a). Therefore, we investigated the involvement of FGF2/FGFR1 paracrine activation by estrogen-stimulated CAFs on CTGF expression in MDA-MB-231 cells. Worthy of note, CM collected from E2 and G-1 treated-CAFs triggered CTGF expression at both mRNA (Figure 6b) and protein levels (Figure 6c,d) in FGFR1 (WT) MDA-MB-231 cells but not in FGFR1 (KO) MDA-MB-231 cells. Next, the up-regulation of CTGF protein levels observed in the aforementioned conditions was also abrogated in the presence of the FGFR1 inhibitor PD173074, the MEK inhibitor PD98059 as well as the PI3K inhibitor Wortmannin (WM) (Figure 6e–g). Altogether, these findings suggest that FGF2/FGFR1 paracrine activation induced by estrogen-stimulated CAFs prompts CTGF expression through the involvement of ERK1/2 and AKT signaling cascades in MDA-MB-231 cells.

### 3.5. FGF2/FGFR1 Paracrine Activation Induces Cell Migration and Invasion Through CTGF in MDA-MB-231 Cells

**Figure 7:**
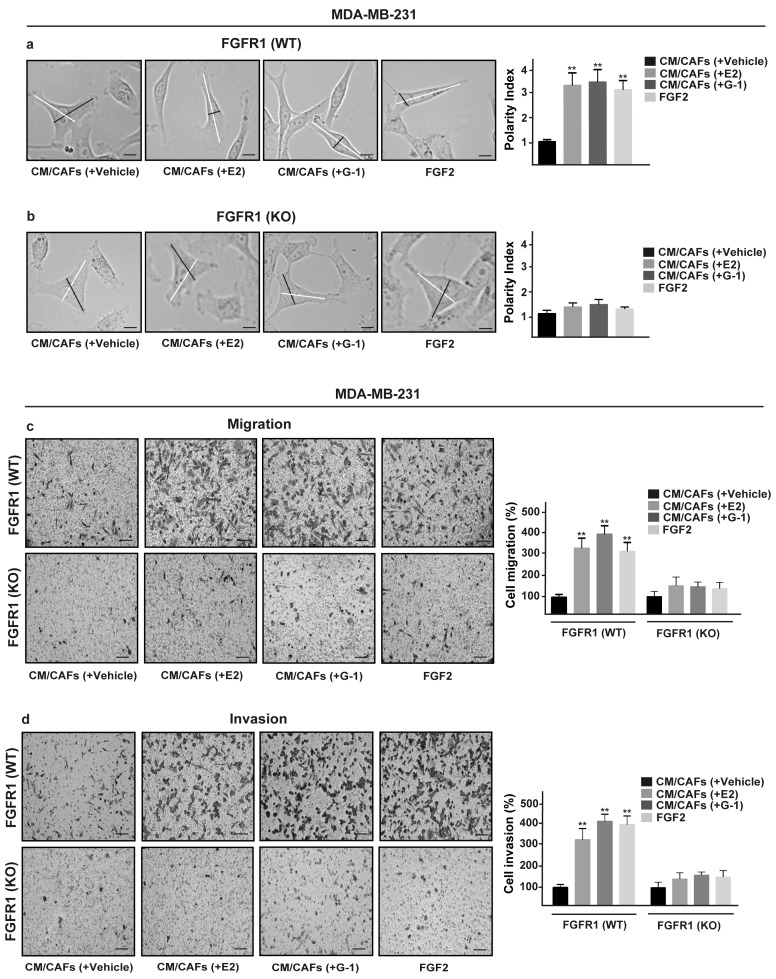
FGFR1 paracrine activation promotes migration and invasion in MDA-MB-231 cells. (**a**) FGFR1 (WT) and **(b**) FGFR1 (KO) MDA-MB-231 cells were cultured for 8 h in CM from CAFs treated for 18 h with vehicle [CM/CAFs (+vehicle)], 10 nM E2 [CM/CAFs (+E2)] or 100 nM G-1 [CM/CAFs (+G-1)], or exposed to 25 nM FGF2, as positive control. Lines traced on cells were used to calculate the Polarity Index (PI). White lines define the migratory axis and black lines the transversal axis. PI = 1.0 indicates a polygonal shape, whereas a value > 1.0 defines ranges of migratory shapes. Scale bar = 30 μm. Images shown are representative of 30 random fields acquired in three independent experiments. Transwell assays were used to assess cell migration (**c**) and invasion (**d**) in FGFR1 (WT) and FGFR1 (KO) MDA-MB-231 cells cultured for 8 h in CM from CAFs treated for 18 h with vehicle [CM/CAFs (+vehicle)], 10 nM E2 [CM/CAFs (+E2)] or 100 nM G-1 [CM/CAFs (+G-1)], or exposed to 25 nM FGF2, as positive control. Cells were counted in at least 10 random fields at 10× magnification, in three independent experiments performed in triplicate. Scale bar = 200 μm, (**) indicates *p* < 0.01.

**Figure 8:**
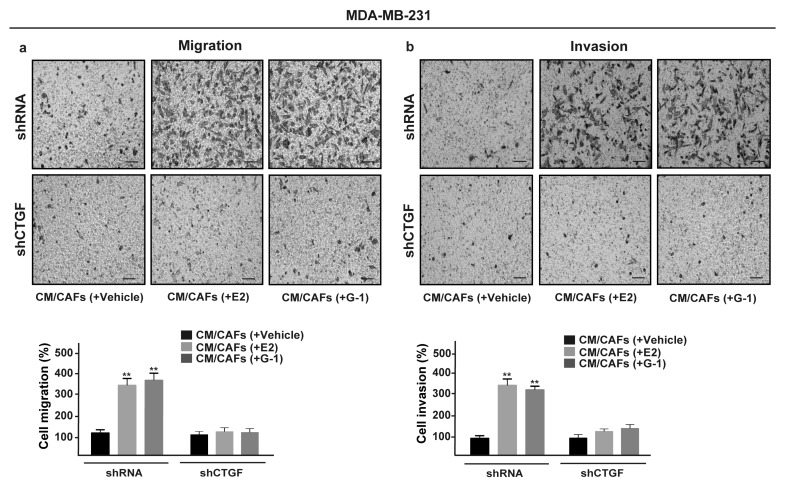
CTGF is required for migration and invasion induced by FGFR1 paracrine activation in MDA-MB-231 cells. Transwell assays were used to assess cell migration (**a**) and invasion (**b**) in MDA-MB-231 cells transfected for 24 h with control shRNA or shCTGF and then cultured for 8 h in CM from CAFs treated for 18 h with vehicle [CM/CAFs (+vehicle)], 10 nM E2 [CM/CAFs (+E2)] or 100 nM G-1 [CM/CAFs (+G-1)]. Cells were counted in at least 10 random fields at 10× magnification, in three independent experiments performed in triplicate. Scale bar = 200 μm, (**) indicates *p* < 0.01.

Upon FGF2/FGFR1 activation, breast cancer cells may acquire invasive phenotype features modulating the expression of cell junction proteins, promoting a spindle-like morphology and increasing cell motility [52,53,54]. Recapitulating the abovementioned results, CM collected from E2 and G-1 treated-CAFs increased spindle-like morphology in FGFR1 (WT) MDA-MB-231 cells, but not in FGFR1 (KO) MDA-MB-231 cells as evaluated by the polarity index (Figure 7a,b). Performing scratch (Supplementary Figure S5a,b) and transwell assays (Figure 7c,d), we then observed that the migration and invasion of FGFR1 (WT) MDA-MB-231 cells promoted by CM from E2 and G-1 treated-CAFs were no longer evident in FGFR1 (KO) MDA-MB-231 cells as well as using the FGFR1 inhibitor PD173074 (data not shown). Next, we found that, in MDA-MB-231 cells, CTGF silencing prevents the migratory and invasive effects triggered by CM obtained from E2 and G-1 treated-CAFs (Figure 8a,b). Taken together, these results suggest that CTGF is involved by FGF2/FGFR1 paracrine activation toward important biological features elicited in MDA-MB-231 cells.

## 4. Discussion

In the current study, we provide novel evidence regarding the role of GPER in the regulation of FGF2 expression triggered by estrogens within the tumor microenvironment. In particular, using primary patient-derived breast CAFs, we ascertained that both E2 and the selective GPER agonist G-1 induce the expression and secretion of FGF2 activating the GPER/EGFR/ERK/c-fos/AP-1 signaling cascade. Analyzing publicly available databases, we then showed that FGFR1 is the most frequently amplified receptor of the FGFRs family along with its association with shorter survival rates in breast cancer patients [38,39,40,41]. In addition, focusing on the FGF2/FGFR1 functional interaction that occurs between CAFs and breast cancer cells, we determined that FGF2 secretion by estrogens-treated CAFs prompts the up-regulation of CTGF expression through the FGFR1-ERK1/2-AKT signaling cascade in MDA-MB-231 cells. As biological counterpart, we found that cell motility and invasiveness triggered by the FGF2/FGFR1 paracrine activation are abrogated by CTGF silencing.

In recent years, considerable attention has been deserved to the involvement of the tumor stroma toward cancer development [55]. In this regard, it has been shown that the interactions between tumor cells and the associated stroma represent a solid relationship that impacts disease initiation, progression, and patient prognosis [56]. For instance, CAFs acting as main players within the tumor stroma, provide a supportive microenvironment for aggressive features of cancer cells [57,58,59,60]. Indeed, CAFs are able to sustain cancer cell growth together with the invasion and metastasis via paracrine actions elicited by cytokines and growth factors released in the tumor microenvironment [6,61]. To date, in breast malignancies ~80% of stromal fibroblasts acquire the activated landscapes of CAFs that boost the proliferation of cancer cells at both the primary and the metastatic sites [62]. Additionally, CAFs may increase the in situ estrogen production, which contributes to the development of breast carcinomas through a multifaceted interactions among different transduction pathways [18,63]. In this vein, several lines of evidence have shown that cancer cells may acquire aberrant growth and invasion properties through the dysregulation of the FGF/FGFR signaling [8,64], as highlighted in large-scale analyses of human cancer genomes [65,66,67,68]. Moreover, the up-regulation and secretion of FGF2 toward the stimulation of FGFR1 signaling in breast cancers was reported to occur upon estrogen stimulation through the classical ER [32,43]. GPER has been also involved in the stimulatory effects exerted by estrogens and its expression was associated with the tumor size, the distant metastasis, and the recurrence of breast malignancies [21,22,23]. Likewise, the role of GPER has been ascertained in CAFs toward the proliferation, migration, and spreading of breast tumor cells [22]. In accordance with these findings, our current results provide new data showing that GPER mediates the expression and secretion of FGF2 in breast CAFs leading to the paracrine activation of the FGFR1-ERK1/2-AKT transduction signaling along with important biological responses in MDA-MB-231 cells.

Metastasis, the leading cause of mortality for breast cancer patients, is a complex and multi-stage process that comprise cellular transformation and tumor growth, angiogenesis, and invasion of target organs [69,70]. In this context, it has been reported that EMT may prompt diverse processes of the metastatic cascade [71,72]. Accordingly, recent studies have shown that FGFR1 activation promotes EMT and metastasis through different signaling pathways in various tumors as prostate, breast, and lung cancers [17,46,73]. High FGF2 expression and secretion have been found in triple-negative breast cancer cell lines, in particular in those showing a mesenchymal phenotype [47,74]. In addition, several factors including vascular endothelial growth factor (VEGF), PC-cell-derived growth factor (PCDGF), epidermal growth factor (EGF), and CTGF were demonstrated to confer migratory and metastatic properties to breast cancer cells [75,76]. As CTGF is concerned, mechanical stresses, cytokines, and growth factors stimulations have been reported to be able to alter its expression levels toward the cytoskeletal reorganization and migratory features in breast cancer cells [24,75,77,78]. Previous studies have also suggested a correlation between FGF2 levels and CTGF-activated signaling in different pathophysiological conditions [49,50,51]. Further corroborating these findings, in the present study a positive correlation between FGFR1 and CTGF expression was assessed by a bioinformatic analysis on 1904 breast tumor samples retrieved from METABRIC dataset. Next, we demonstrated that the paracrine activation of the FGF2/FGFR1 transduction pathway prompts the expression of CTGF, which was involved in the migratory and invasive responses observed in MDA-MB-231 cells.

## 5. Conclusions

Our findings indicate that GPER mediates a feed-forward FGF2/FGFR1 engagement within the tumor microenvironment linking CAFs to breast cancer cells toward tumor progression. Moreover, on the basis of the present data GPER may be included among the transduction mechanisms involved in the FGF2/FGFR1 paracrine activation that may contribute to breast cancer development.

## Supplementary Materials

The following are available online at https://www.mdpi.com/2073-4409/8/3/223/s1, Figure S1. Characterization of primary cultured CAFs and NFs; Figure S2. Efficacy of GPER silencing; Figure S3. Up-regulation of FGF2 expression by E2 and G-1 is mediated by the GPER-EGFR-ERK1/2 transduction pathway in CAFs; Figure S4. mRNA expression of FGF2 and FGFR1 in MDA-MB-231, SkBr3 and MCF-7 cells; Figure S5. Scratch assay upon exposure to CM from estrogen-stimulated CAFs in MDA-MB-231 cells.

## Abbreviations

CAFs	cancer associated fibroblasts
FGFR1	fibroblast growth factor receptor 1
FGF2	fibroblast growth factor 2
GPER	G protein estrogen receptor
CRISPR	Clustered Regularly Interspaced Short Palindromic Repeats
sgRNA	single guide RNA
WT	Wild type
KO	Knockout ERK, extracellular signal-regulated kinase
AP-1	activator protein 1
PI3K	phosphatidylinositol 3-kinase
CTGF/CCN2	connective tissue growth factor
