# Supplementary material for: GPER Mediates a Feedforward FGF2/FGFR1 Paracrine Activation Coupling CAFs to Cancer Cells toward Breast Tumor Progression
Source: Cells. 2019 Mar 7;8(3):223. doi: 10.3390/cells8030223 (PMC6468560; doi:10.3390/cells8030223)
Supplement: Supplementary file 1 [file cells-08-00223-s001.zip › sup/Supplementary Figures with figure legends.docx]

**Supplementary figures and figure legends**

**
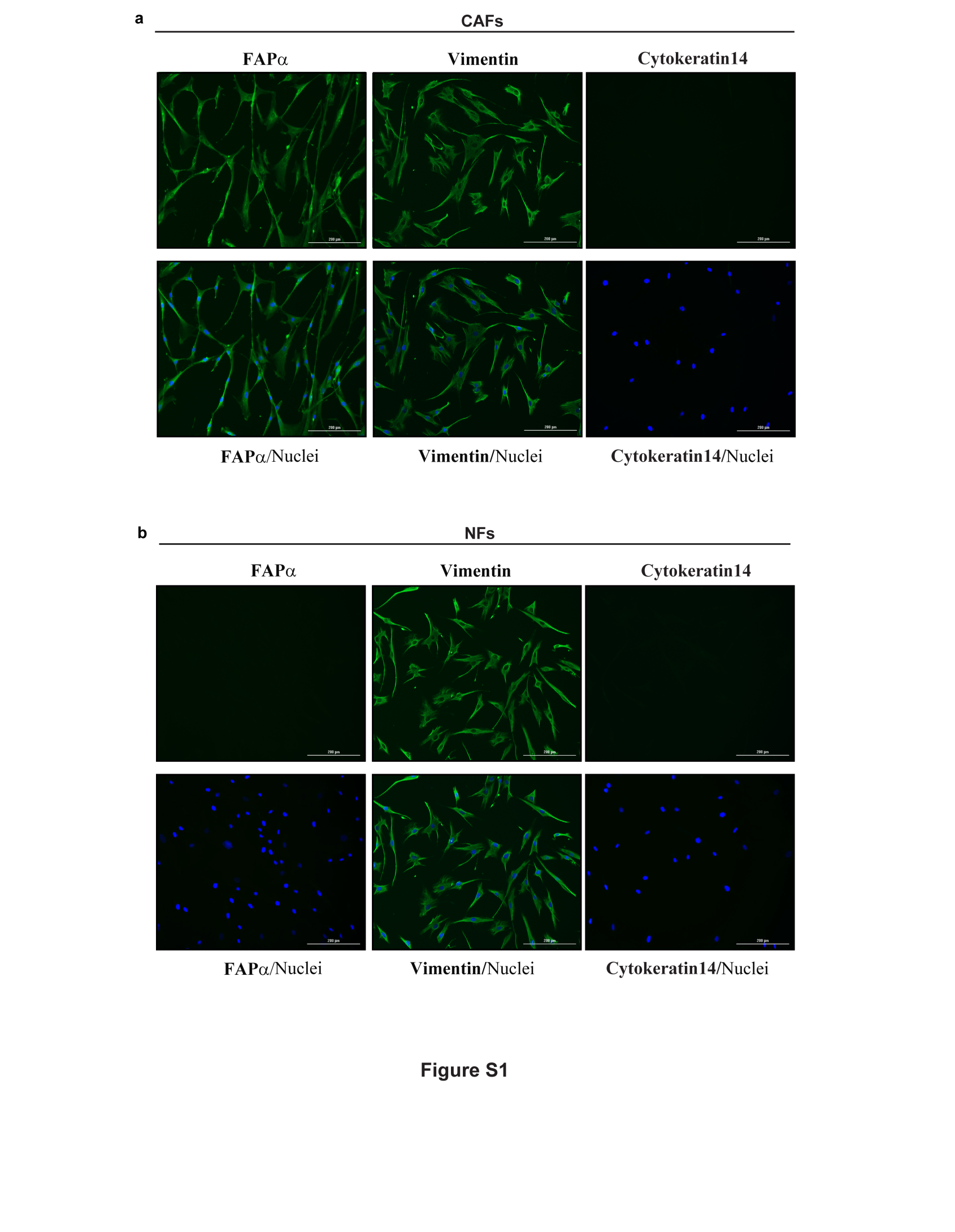
**

**Figure S1. Characterization of primary cultured CAFs and NFs.** CAFs (**a**) and NFs (**b**) were immunostained by anti-FAPα, anti-Vimentin and anti-Cytokeratin14 antibodies. *Green signal*: FAPα and Vimentin; *blue signal*: nuclei (DAPI). Scale bar: 200 μm.

**

**

**Figure S2. Efficacy of GPER silencing.** β-actin served as a loading control. Side panels show densitometric analysis of the blots normalized to the loading control. Immunoblots shown are representative of three independent experiments.


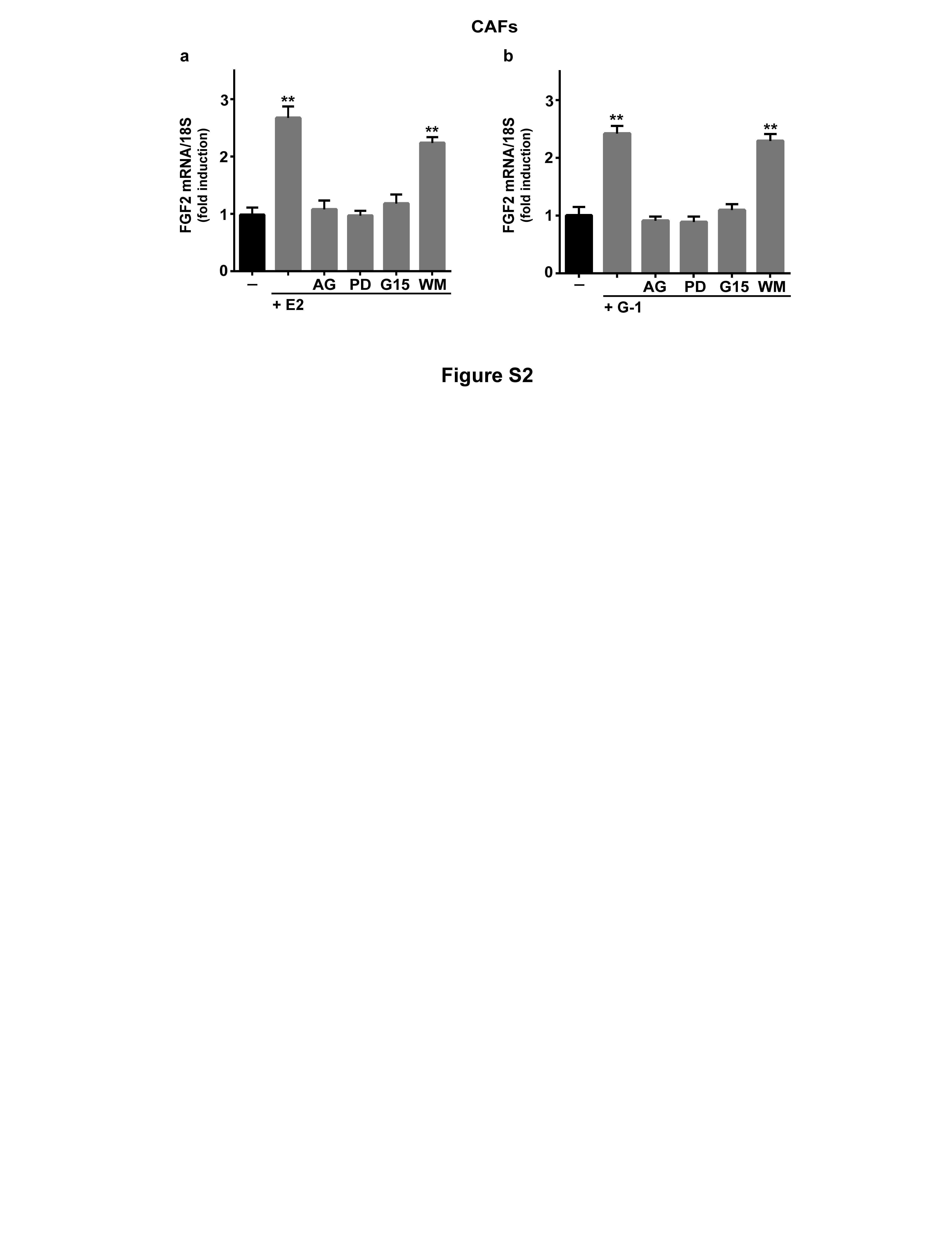


**Figure S3. Up-regulation of FGF2 expression by E2 and G-1 is mediated by the GPER-EGFR-ERK1/2 transduction pathway in CAFs. (a, b)** Up-regulation of FGF2 mRNA expression observed in CAFs treated for 3 h with 10 nM E2 or 100 nM G-1 was abolished by 1 µM EGFR inhibitor AG1478 (AG), 10 μM MEK inhibitor PD98059 (PD) or 100 nM GPER antagonist G15, whereas 100 nM of the PI3K inhibitor Wortmannin (WM) was ineffective. Values were normalized to 18S expression and shown as fold changes of FGF2 mRNA expression upon treatments respect to cells exposed to vehicle (-). Each column represents the mean ± SD of three independent experiments performed in triplicate. (**) indicates p< 0.01.


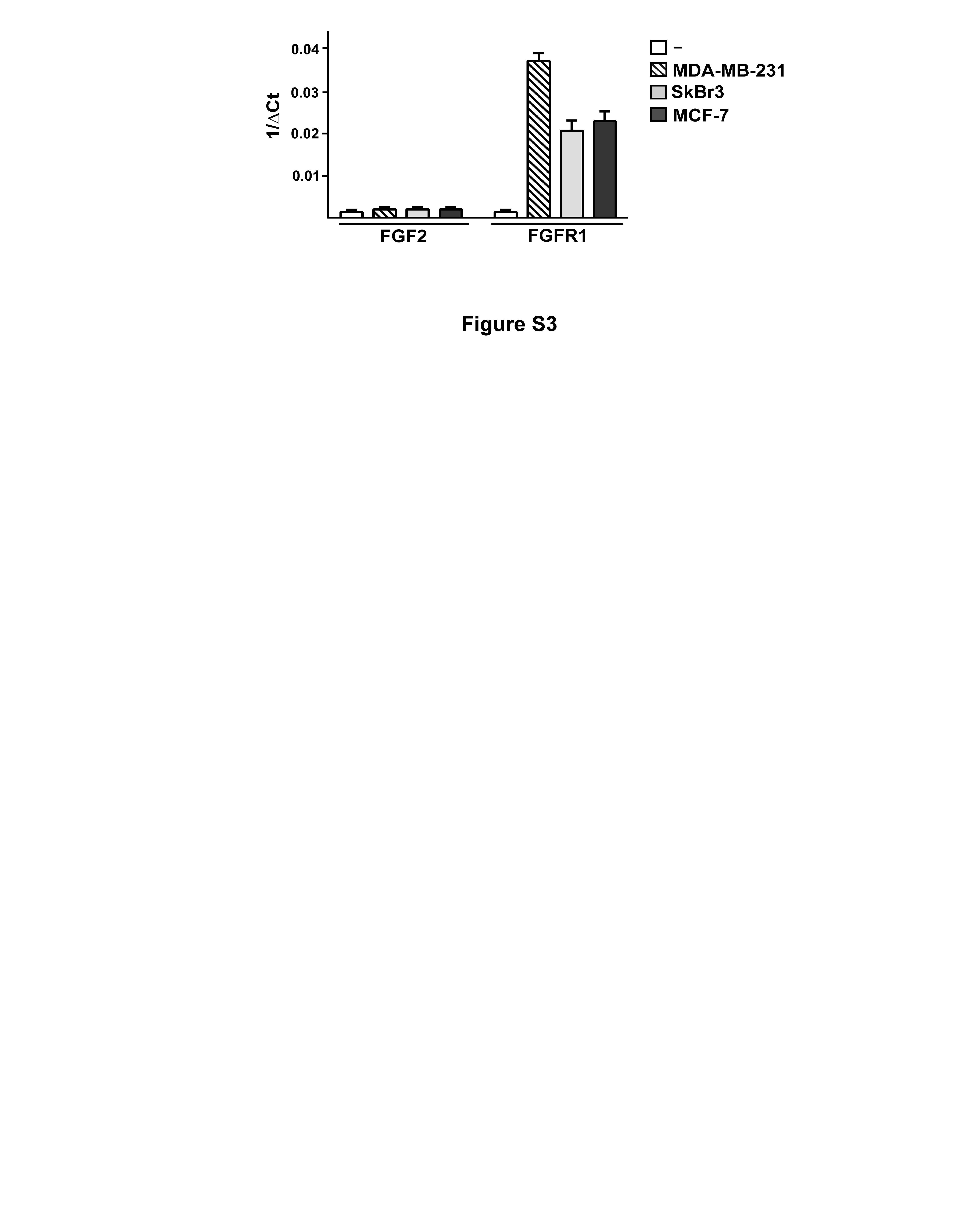


**Figure S4. mRNA expression of FGF2 and FGFR1 in MDA-MB-231, SkBr3 and MCF-7 cells.** PCR amplification in the absence of cDNA was used as a negative control (-). Each column represents the mean ± SD of three independent experiments performed in triplicate.


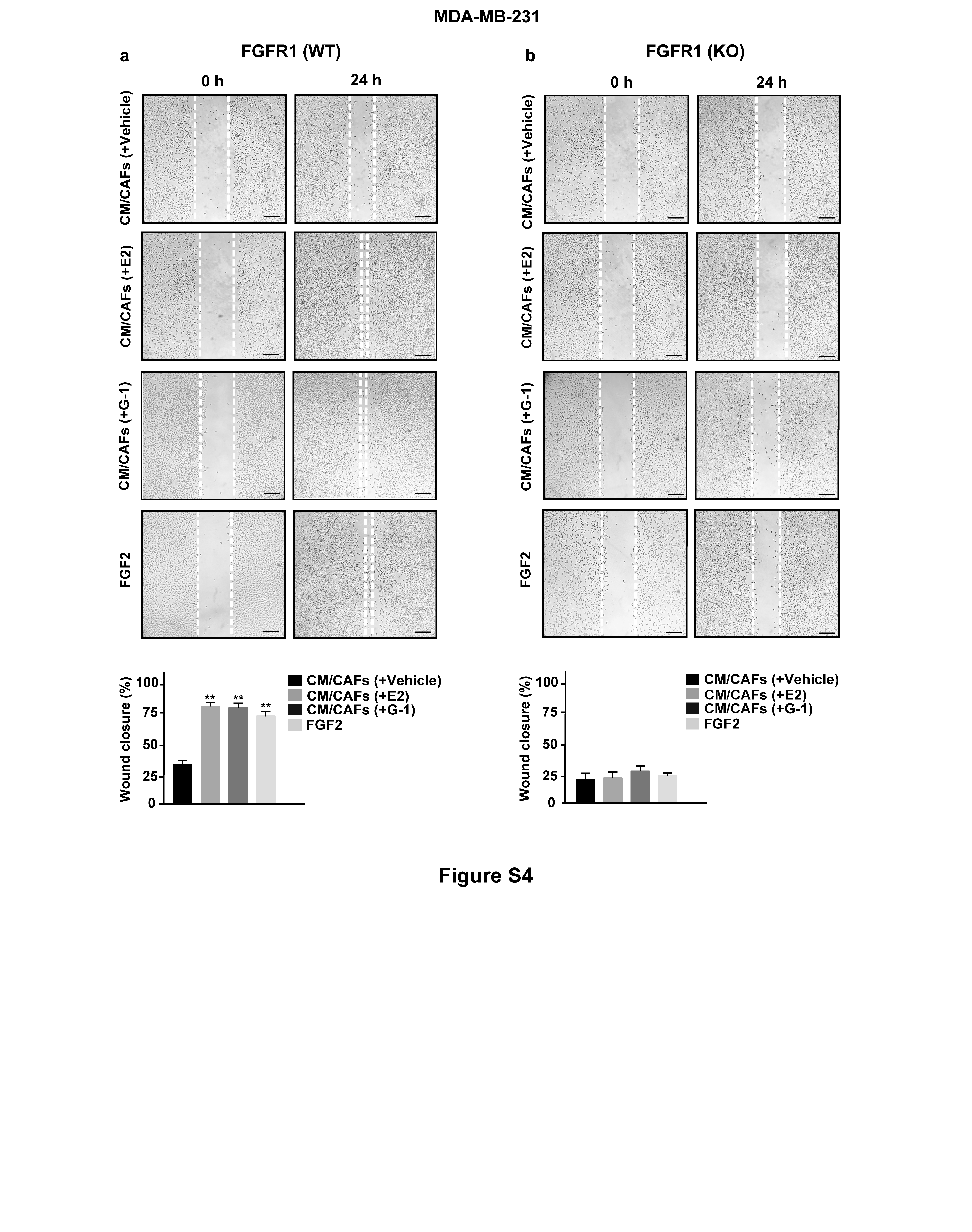


**Figure S5. Scratch assay upon exposure to CM from estrogen-stimulated CAFs in MDA-MB-231 cells.** FGFR1 (WT) (**a**) and FGFR1 (KO) (**b**) MDA-MB-231 cells were incubated for 24 h with CM from CAFs treated for 18 h with vehicle [CM/CAFs (+vehicle)], 10 nM E2 [CM/CAFs (+E2)] or 100 nM G-1 [CM/CAFs (+G-1)], or with 25 nM FGF2, as positive control. Images were acquired at 0 h and 24 h after scratching. Quantification of cell migration was expressed as % of wound closure. Scale bar= 400 μm. Data shown are the mean ± SD of three independent experiments performed in triplicate. (**) indicates p< 0.01.
