# Supplementary figures and images for: GPER Mediates a Feedforward FGF2/FGFR1 Paracrine Activation Coupling CAFs to Cancer Cells toward Breast Tumor Progression
Source: Cells. 2019 Mar 7;8(3):223. doi: 10.3390/cells8030223 (PMC6468560; doi:10.3390/cells8030223)

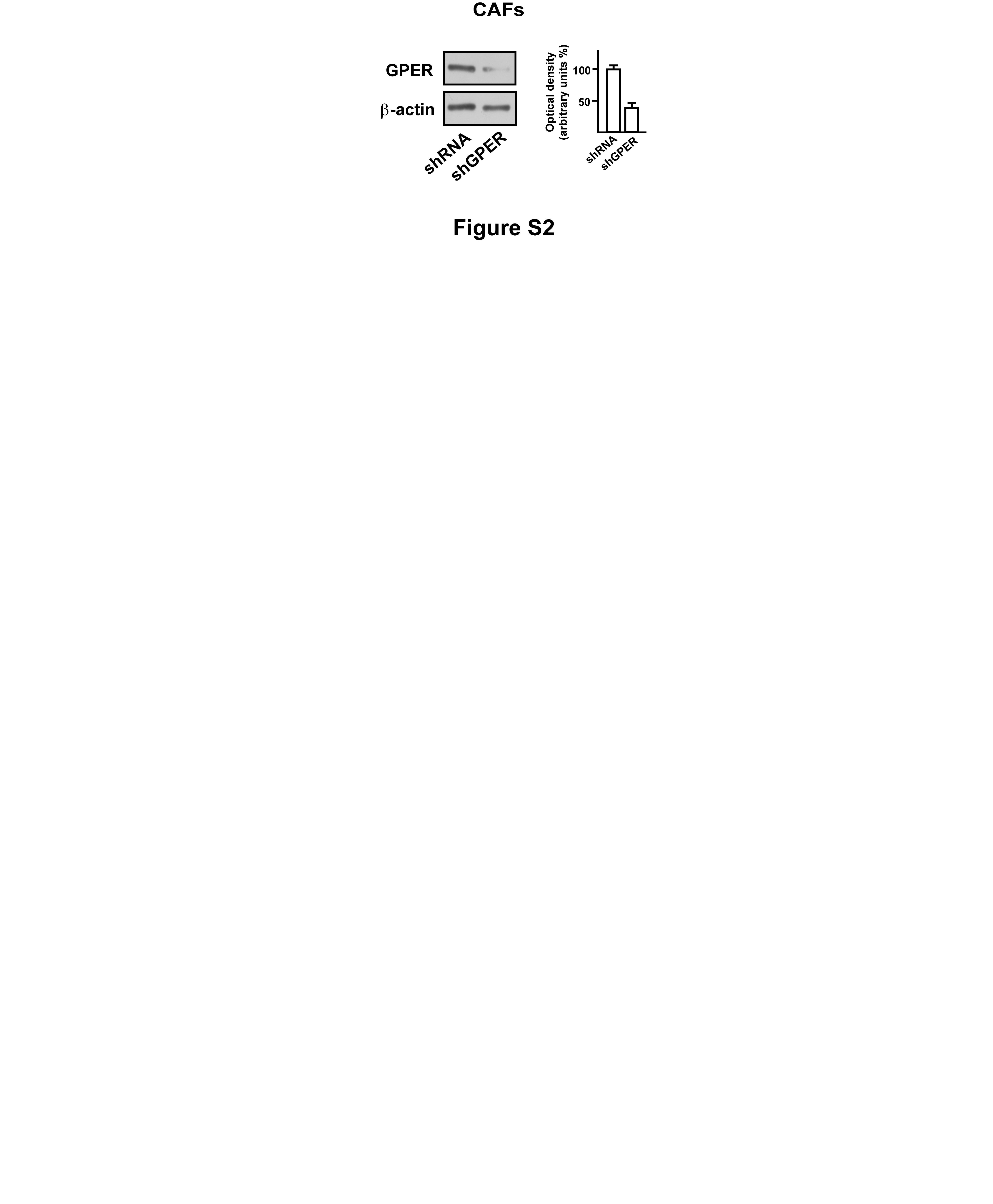

Supplement: Supplementary file 1 [file cells-08-00223-s001.zip › sup/Figure S2.tif]

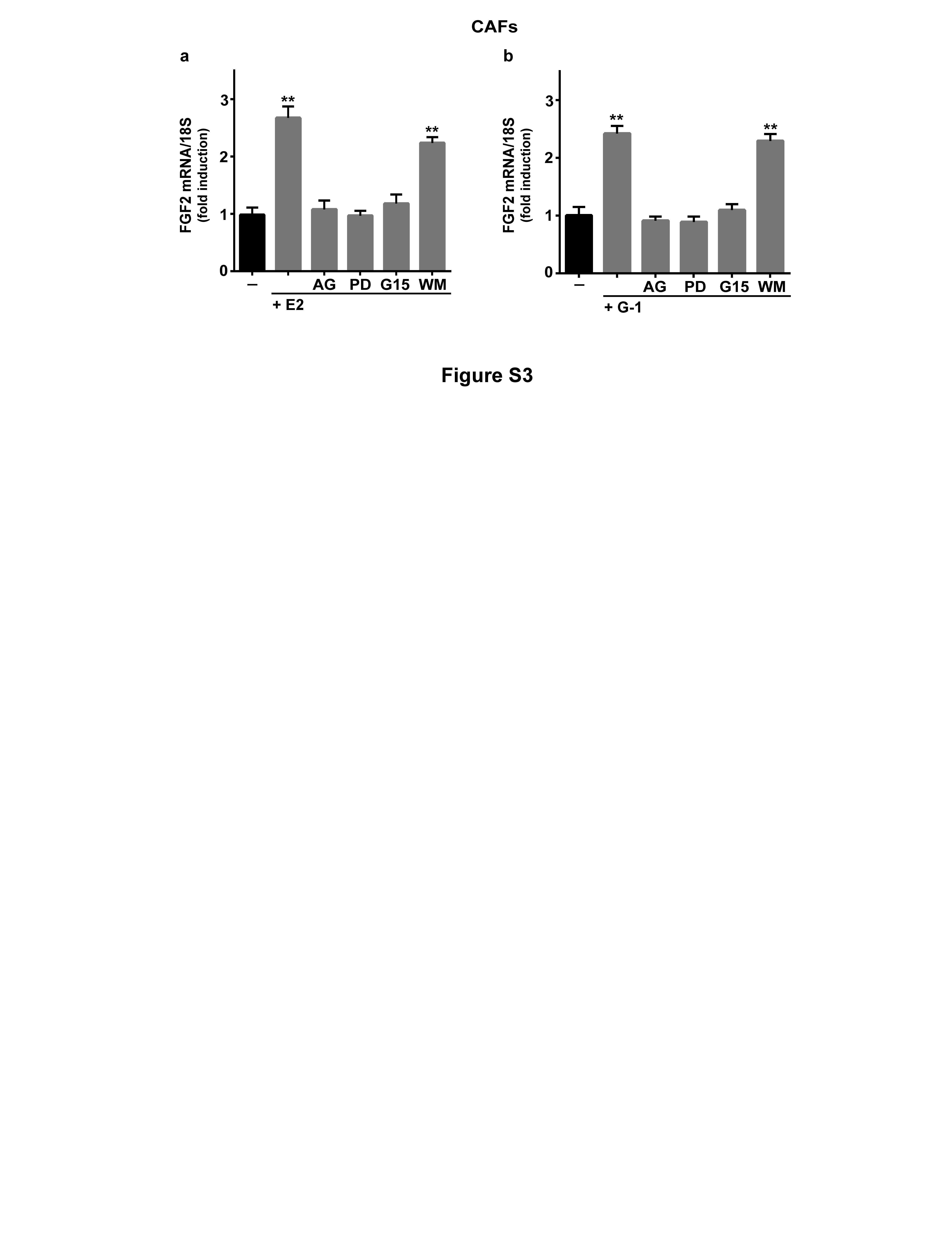

Supplement: Supplementary file 1 [file cells-08-00223-s001.zip › sup/Figure S3.tif]

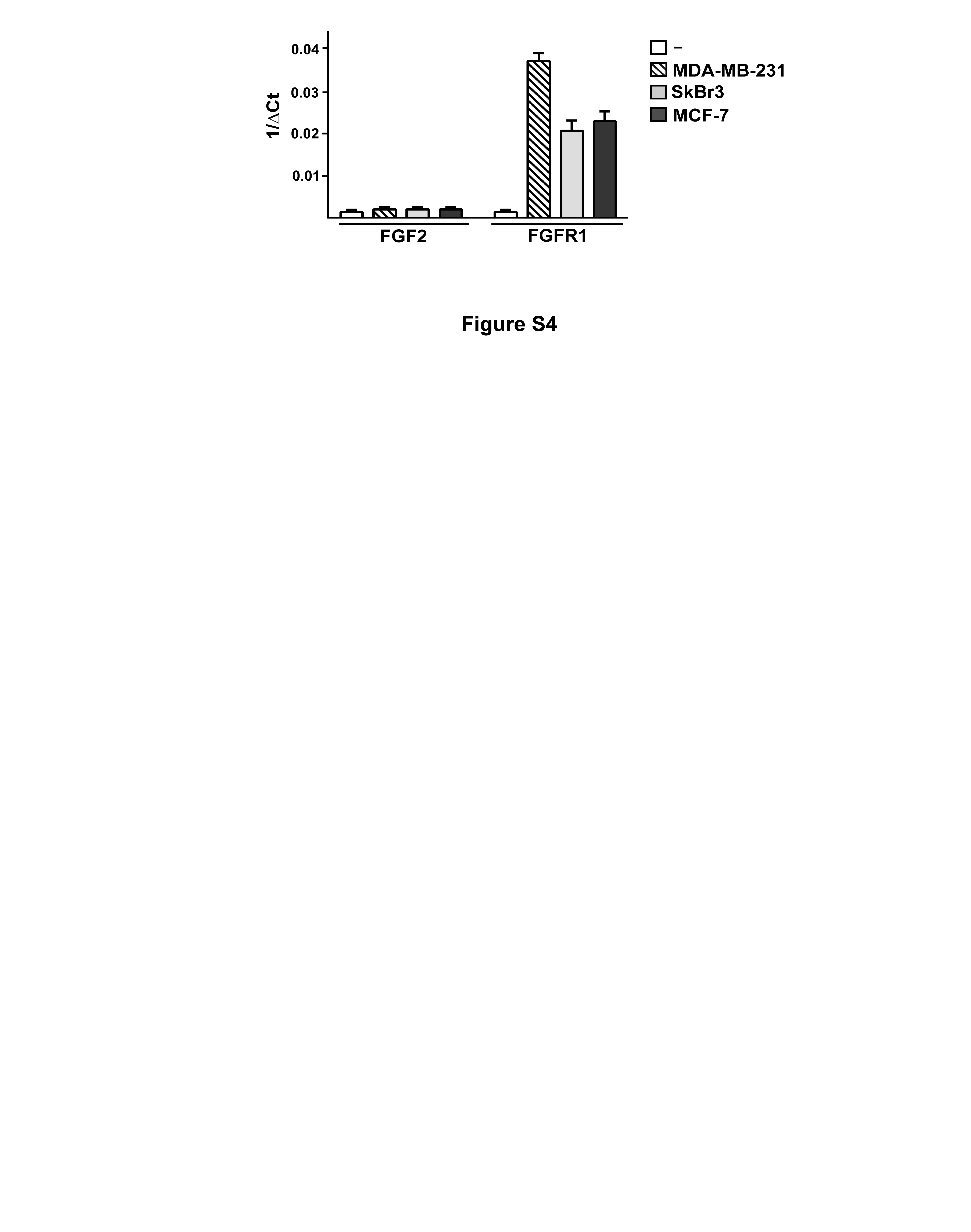

Supplement: Supplementary file 1 [file cells-08-00223-s001.zip › sup/Figure S4.tif]

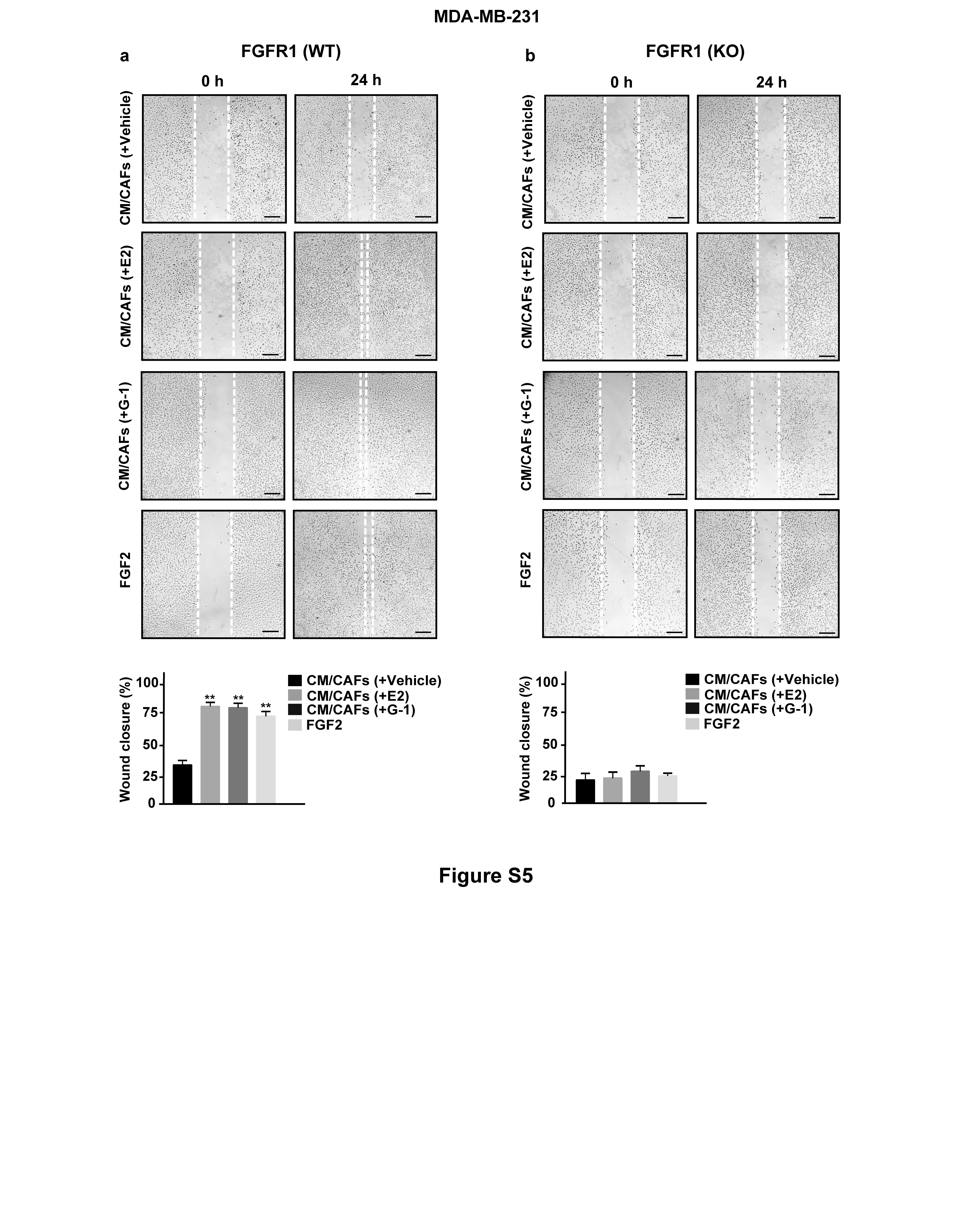

Supplement: Supplementary file 1 [file cells-08-00223-s001.zip › sup/Figure S5.tif]
